# Graphene Oxide Normal (GO + Mn^2+^) and Ultrapure: Short-Term Impact on Selected Antioxidant Stress Markers and Cytokines in NHDF and A549 Cell Lines

**DOI:** 10.3390/antiox10050765

**Published:** 2021-05-11

**Authors:** Dominika Stygar, Aleksandra Pogorzelska, Elżbieta Chełmecka, Bronisława Skrzep-Poloczek, Barbara Bażanów, Tomasz Gębarowski, Jerzy Jochem, Jiří Henych

**Affiliations:** 1 Department of Physiology in Zabrze, Faculty of Medical Sciences in Zabrze, Medical University of Silesia, Poniatowskiego 15 Street, 40-055 Katowice, Poland; rehabilitacja-kopernik@o2.pl (B.S.-P.); jjochem@poczta.onet.pl (J.J.); 2 Division of Microbiology, Faculty of Veterinary Medicine, Wroclaw University of Environmental and Life Sciences, Norwida 31 Street, 50-375 Wrocław, Poland; barbara.bazanow@upwr.edu.pl (B.B.); aleksandra.pogorzelska@upwr.edu.pl (A.P.); 3 Department of Statistics, Department of Instrumental Analysis, Faculty of Pharmaceutical Sciences in Sosnowiec, Medical University of Silesia, Ostrogórska 30 Street, 40-055 Katowice, Poland; echelmecka@sum.edu.pl; 4 Department of Basic Medical Sciences, Faculty of Pharmacy, Wroclaw Medical University, Borowska 211 Street, 50-367 Wrocław, Poland; tomasz.gebarowski@umed.wroc.pl; 5 Institute of Inorganic Chemistry, Czech Academy of Sciences, 250 68 Husinec-Řež, Czech Republic; henych@iic.cas.cz

**Keywords:** A549, NHDF, GO, cytokines, graphene-based materials, graphene oxide, oxidative stress markers

## Abstract

Since biological applications and toxicity of graphene-based materials are structure dependent, studying their interactions with the biological systems is very timely and important. We studied short-term (1, 24, and 48 h) effects of ultrapure (GO) and Mn^2+^-contaminated (GOS) graphene oxide on normal human dermal fibroblasts (NHDF) and adenocarcinomic human alveolar basal epithelial cells (A549) using selected oxidative stress markers and cytokines: glutathione reductase (GR) and catalase (CAT) activity, total antioxidative capacity (TAC), and malondialdehyde (MDA) concentration, levels of vascular endothelial growing factor (VEGF), tumor necrosis factor-alpha (TNF-α), platelet-derived growth factor-BB (PDGF-BB), and eotaxin. GOS induced higher levels of oxidative stress, measured with CAT activity, TAC, and MDA concentration than GO in both cell lines when compared to control cells. GR activity decreased in time in NHDF cells but increased in A549 cells. The levels of cytokines were related to the exposure time and graphene oxide type in both analyzed cell lines and their levels comparably increased over time. We observed higher TNF-α levels in NHDF and higher levels of VEGF and eotaxin in the A549 cell line. Both types of cells showed similar susceptibility to GO and GOS. We concluded that the short-time exposure to GOS induced the stronger response of oxidative stress markers without collapsing the antioxidative systems of analysed cells. Increased levels of inflammatory cytokines after GO and GOS exposure were similar both in NHDF and A549 cells.

## 1. Introduction

Rapid progress in the production of diverse, graphene-based nano-composites makes studying their interaction with the biological systems a very important topic. We know that their biological applications depend on the synthesis method and their functionalization. We also know that the toxicological effects of graphene-based materials are structure dependent and may vary substantially [1]. Graphene oxide (GO) is widely studied in the context of biosensors [2], its antibacterial properties, tissue regenerative capacities [3], and drug delivery possibilities [4]. Unfortunately, a considerable number of sources describe graphene oxide’s toxicity within animal models and cell cultures. In animals, lung granuloma formation, liver and kidney injuries, and decreased cell viability represent some of many undesirable effects of GO’s dose-dependent toxicity [5,6,7,8]. Graphene toxicity at the cellular level mainly affects cell viability, morphology, integrity, mitochondrial activity, and DNA structure. Its presence promotes the generation of reactive oxygen species (ROS), which eventually leads to oxidative stress (OS). Moreover, as a highly oxidized form of graphene, GO can easily interact with different biomolecules [9].

Oxidative stress causes disproportion between ROS production and antioxidant defenses and disrupts cellular physiology [10] because enzymatic and non-enzymatic antioxidant molecules should be maintained at a physiological level [11] including the amount and activity of catalase (CAT) and glutathione reductase (GR) [12]. Malondialdehyde (MDA) is one of the final products of polyunsaturated fatty acids’ peroxidation in the cells and it is overproduced in the presence of high levels of ROS [13]. These enzymatic and non-enzymatic indicators together with more comprehensive parameters, like total antioxidant capacity (TAC), help to assess and understand the sensitive balance between oxidants and antioxidants in vivo and in vitro [14].

Cytokines are considered good inflammatory indicators that may be used to assess health and disease status. The accurate profiling of selected multiple cytokines is substantial to get a comprehensive and thorough understanding of the complex physiological and pathological conditions of the cells [15], hopefully also these caused by graphene and its derivatives. Despite the high probability of the toxic effects of GO, so far only a few studies have addressed the effects of GO on the levels of oxidative stress markers and cytokines in in vitro cultures [16,17,18,19].

Our study aimed to verify the possible effect of GO quality on the oxidative stress and cytokine levels in in vitro cultures. We studied GR and CAT activity, TAC, and MDA concentration as good markers of oxidative stress, and selected cytokines (vascular endothelial growing factor (VEGF), tumor necrosis factor-alpha (TNF-α), platelet-derived growth factor-BB (PDGF-BB), and eotaxin) as good indicators of inflammatory conditions. We compared the biological effects of two types of GO, ultrapure (GO) and contaminated with Mn2+ ions (GOS), on two types of cell lines, normal human dermal fibroblasts (NHDF) and adenocarcinomic human alveolar basal epithelial cells (A549), expecting different scopes of biological response. With this experimental setup, we aimed to assess the effects of GO derivatives at the cellular level after a short-term exposure (1, 24, and 48 h), looking for a relatively good GO exposure marker among the selected oxidative stress markers and cytokines.

## 2. Materials and Methods

### 2.1. Graphene Oxide Synthesis

All chemicals were obtained from Sigma-Aldrich (Czech Republic) and were used without further purification. Both graphene oxide samples were prepared from natural graphite (Koh-i-noor Hardmuth, Czech Republic). Graphene oxide and GO-contaminated materials were characterized and described by Zhao et al. [20]. The ultrapure GO was characterized in detail by Štengl [21], Ederer et al. [22,23], and Ahlinder et al. [24].

#### 2.1.1. Graphene Oxide Manganese Ion Contaminated (GO + Mn^2+^, GOS)

Graphene oxide manganese ion contaminated (GO + Mn^2+^), here called GOS, was prepared using the modified Hummers method [20]. Graphite (4.0 g) and NaNO3 (3.0 g) were mixed with H_2_SO_4_ (300 mL) and stirred in an ice-water bath. Then, KMnO_4_ (18.0 g) was slowly added in several portions and the mixture was continuously stirred for 5 d at room temperature. Next, the mixture was heated to 98 ± 1 °C and 5 wt% H_2_O_2_ (560 mL) was added in small portions in the course of 2 h. The suspension was stirred for the next 2 h at 98 ± 1 °C. Then, the mixture was left to cool down to 60 °C and 30 wt% H_2_O_2_ (12 mL) was added to the suspension and further stirred for another 2 h at room temperature. The obtained mixture was washed with water and centrifuged at 5000 rpm five times. Finally, the precipitate was rinsed with Milli-Q water until the supernatant solution was neutral.

#### 2.1.2. Graphene Oxide Ultrapure (GO)

The ultrapure GO was prepared using a slightly modified procedure that is based on high-power ultrasonication in a pressurized water-cooled reactor and modified Hummers oxidation method [20]. Graphite (1 g) was dispersed in ethylene glycol (100 mL) and sonicated for 60 min in a water-cooled stainless steel reactor (UiP100hd, Hielscher Ultrasonics, Germany) using the ultrasonic horn (20 kHz, 1000 W) under overpressure of 600 kPa. The obtained mixture was purified in distilled water using dialysis membrane (Spectra/Por 3, Thermo Fisher Scientific, Waltham, MA, USA) and then the excess water was filtered off. Then, the sonicated graphite was carefully mixed with concentrated H_2_SO_4_ (60 mL) and H_3_PO_4_ (10 mL) by slowly stirring in an ice bath. Next, KMnO_4_ (3 g) was gradually added and the obtained mixture was heated to 40 °C and stirred overnight. Then, the formed, brown, viscous liquid was poured onto the ice with 30% H_2_O_2_ (100 mL). Next, the obtained yellowish suspension was thoroughly washed with water and centrifuged at 5000 rpm (until water pH = 3.5–4), followed by purging in distilled water by dialysis under mild sonication (ultrasonic bath, 300 W, 4 h). This last step efficiently removed any residual Mn^2+^, SO_4_^2−^, and PO_4_^3−^ ions from the synthesis, and the dark brown suspension of ultrapure GO was obtained.

### 2.2. Cell Culture

In vitro experiments were carried out using normal human dermal fibroblasts (NHDF, ATTC cat. no CCL-10TM) and adenocarcinomic human alveolar basal epithelial cells (A549, ATCC cat. no CCL-37TM) (American Type Culture Collection, Manassas, VA, USA).

Cells were grown in Dulbecco’s Modified Eagle’s Medium (DMEM) with non-essential amino acids. Cultures were maintained in Corning culture flasks (75 cm^3^) at 37 °C in an incubator supplied with 5% CO_2_ and 95% air humidity. When the cells reached confluence of 90%, they were split using 0.25% trypsin-EDTA and distributed evenly into new flasks. The cells were allowed to attach to the surface for 24 h before GO/GOS treatment.

GO/GOS were suspended in Milli-Q water to prepare the stock solution (1 mg/mL). The stock solution was sonicated for 1 h (40 kHz, 50 W) and diluted to 40 μg/mL concentration with DMEM just before cell culture exposure.

For biochemical assays, NHDF and A549 cells were cultured in large flasks and treated with GO and GOS (40 μg/mL) for 1, 24, and 48 h. Cells without any GO/GOS treatment served as controls and were processed identically. After the treatment, the cells were split with trypsin-EDTA and collected in PBS. Then, they were washed several times with PBS at 4 °C and lysed using the lysis buffer (1× 20 mM Tris-HCl, pH 7.5; 150 mM NaCl; 1 mM Na_2_EDTA; 1% Triton; 2.5 mM Na_4_P_2_O_7_). The obtained cellular homogenate was centrifuged at 15,000× *g* (10 min, 4 °C) (Thermo Jouan Centrifuge B4i, Thermo Fisher Scientific, Waltham, MA, USA). Obtained supernatants were stored at −80 °C until biochemical analysis.

### 2.3. GOS and GO Cytotoxic Activity

NHDF and A549 cells (5000 cells/1 mL) were used to determine the cytotoxic dose of GOS and GO. After sonication, GOS and GO were tested using EN 14675 European Standard (chemical disinfectants and antiseptics: quantitative suspension test for the evaluation of the virucidal activity of chemical disinfectants and antiseptics used in veterinary area, test method and requirements (phase 2, step 1)). GOS and GO test solutions were prepared in Minimum Essential Medium (MEM) supplemented with an additional 2% Fetal Bovine Serum (FBS) and L-glutamine. Serial dilutions’ tested complexes (dilution step 1:10 from starting concentration of GOS 8 mg/mL and GO 10.1 mg/mL) were prepared and transferred (50 μL) into cell culture units (wells of microtiter plates) containing suspended cells (50 μL). Eight culture units were inoculated with each dilution. Plates were incubated at 37 °C/ 5% CO_2_ and observed daily for up to 4 d to develop cytotoxic effect (CTE) using an inverted microscope (Olympus Corp., Hamburg, Germany; Axio Observer, Carl Zeiss MicroImaging GmbH, Jena, Germany). The lowest cytotoxic dose of graphene was 68.1 µL/mL in both GO and GOS.

### 2.4. Oxidative Stress Markers Analysis

The oxidative stress markers were analyzed in both cell lines (NHDF, A549) exposed to GOS or GO for 1, 24, and 48 h and respective controls. We determined the activity of the following antioxidant enzymes: glutathione reductase (glutathione-disulfide reductase, GR, GSR) and catalase (CAT). We also analyzed the non-enzymatic antioxidant system by determining the level of total antioxidant capacity (TAC) and the intensity of lipid peroxidation processes by determining malondialdehyde concentration (MDA).

#### 2.4.1. Oxidative Enzymes Analysis

Glutathione reductase (GR) activity [EC 2.5.1.18]

Glutathione reductase (GR) activity was evaluated by measuring NADPH concentration decrease in the GR buffer (200 mM sodium phosphate pH 7.5; 6.3 mM EDTA). The decrease in NADPH concentration was estimated at λ = 340 nm using the kinetic method (10 min) [25].

Catalase (CAT) activity [EC 1.11.1.6]

Catalase (CAT) activity was measured using Aebi kinetic method [26]. The cell homogenate was mixed with TRIS/HCl buffer (50 mM, pH 7.4) and H_2_O_2_ and, after an initial 10 s, the absorbance was read at λ = 240 nm every 30 s, for 2 min. CAT activity was expressed in IU/mg protein [26].

#### 2.4.2. Total Antioxidant Capacity (TAC)

Total antioxidant capacity (TAC) was measured using Randox commercial kit (RandoxCo., UK). In this method, the 2,2 azinodi-(3-ethylbenzothiazoline sulphonate (ABTS) was incubated with a peroxidase (metmyoglobin), hydrogen peroxide, and the sample to produce the radical cation (ABTS+), which had a relatively stable blue-green color (measured at λ = 600 nm). The suppression of the color was then compared to the standard for TAC measurement assays (Trolox, 6-hydroxy-2,5,7,8-tetramethylchroman-2-carboxylic acid). The assay results are expressed as Trolox equivalent (mmol/L) [27].

#### 2.4.3. Lipid Peroxidation

Malondialdehyde (MDA) concentration was assessed according to the Ohkawa method, using the reaction with thiobarbituric acid [28]. The concentration of MDA was detected spectrophotometrically (λ_excitation_ = 515 nm, λ_emission_ = 552 nm wavelengths) and calculated from the standard curve prepared from 1,1,3,3- tetraethoxypropane [28]. The inter- and intra-assay coefficients of variations for this assay were 2.1% and 8.3%, respectively.

### 2.5. Analysis of Pro-Inflammatory Cytokines

The selected cytokines were analyzed in both cell lines (NHDF, A549) exposed to GOS or GO for 1, 24, and 48 h and respective controls. The Human Cytokine Bio-Plex (Hu VEGF, Hu TNF-α, Hu PDGF-BB, Hu Eotaxin) (Bio-Rad, USA) immunoassay was performed with a 96-well, flat-bottom plate following the manufacturer’s guidelines (www.bio-rad.com/bio-plex accessed on 10 May 2021). Briefly, conjugated beads were allowed to react with a sample containing a known (standard) or unknown amount of cytokines for 30 min. Conjugated beads with bound target were then washed and incubated with biotinylated detection antibodies that were directed against specific cytokine epitopes. The formed complexes were then incubated for a further 10 min with streptavidin-phycoerythrin and excess of the reagent was washed off. The results were read using a microtiter plate reader (Bio-Rad). The concentration of cytokines in supernatants was calculated from the generated standard curves for each cytokine using Bio-Plex software (Bio-Rad).

### 2.6. Statistical Analysis

The distribution of variables was evaluated by the Shapiro–Wilk test and a quantile–quantile plot. The homogeneity of variances was assessed by Levene’s test. The interval data were expressed as a mean value ± standard deviation. For data comparison a repeated-measures ANOVA was used. To compare the variances of the differences between all possible pairs of within-subject conditions (i.e., levels of the independent variable) the Mauchly’s tests and the multivariate tests were done. Statistical significance was set at a *p* < 0.05 and all tests were two-tailed. Statistical analysis was performed using the data analysis software system Statistica, version 13.3.0 (TIBCO Software Inc., Palo Alto, CA, USA).

## 3. Results

### 3.1. Oxidative Stress Markers in Cells Exposed to Graphene Oxide Manganese Ion Contaminated (GO + Mn^2+^, GOS) and Graphene Oxide Ultrapure (GO) for 1, 24, and 48 h

#### 3.1.1. NHDF Cell Line

**Figure 1:**
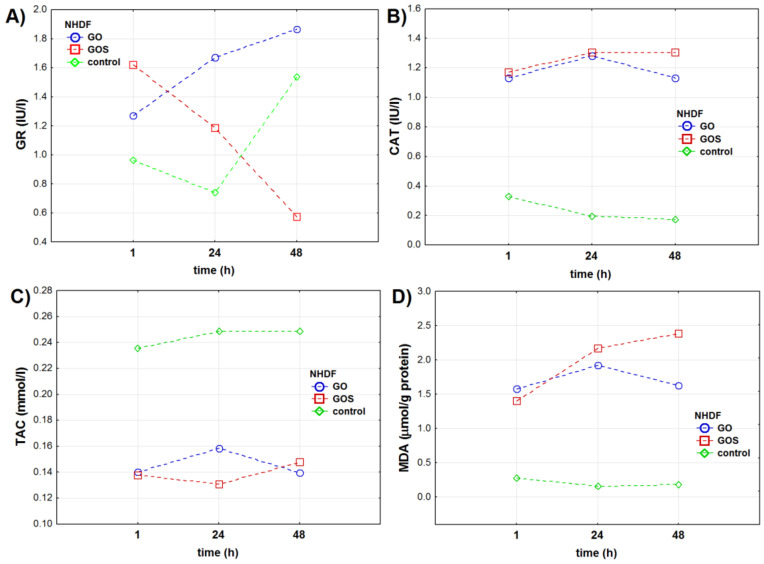
Oxidative stress markers in the NHDF (normal human dermal fibroblasts) cell line measured 1, 24, and 48 h after exposure to ultrapure (GO) or manganese ions-contaminated graphene oxide (GOS): (**A**) glutathione reductase (GR) activity (IU/L), (**B**) catalase (CAT) activity (IU/L), (**C**) total antioxidant capacity (TAC) (mmol/L), (**D**) malondialdehyde (MDA) concentration (μmol/g protein). The markers present mean value of the analyzed parameter.

The analysis of the results showed that glutathione reductase (GR) activity in NHDF (normal human dermal fibroblasts) cells depended on the type of graphene oxide used in the experiment, time of exposure, and a combination of both factors (Table 1, Table S1, and Figure 1a). Over the time, GR activity showed reversed trends in the GO and GOS exposed cells. The highest GR activity was observed after 1 h for GOS-exposed cells and after 24 and 48 h for GO-exposed cells. GOS presence reduced GR activity in NHDF cells after 24 h when compared to control cells and after 48 h when compared to GO-treated and control cells.

Catalase (CAT) activity measured in the NHDF cells depended on the time of the exposure (differently for each graphene type used), but not on GO or GOS itself. After 1 h of the experiment, CAT activity was significantly higher in GO- and GOS-exposed cells when compared to control NHDF cells (Table 1, Table S1, and Figure 1b). After 24 h, CAT activity was the same in GO- and GOS-exposed cells but was higher than in control NHDF cells. After 48 h of the experiment, CAT activity was significantly higher in GOS-exposed cells than in control and GO-exposed NHDF cells.

Total antioxidant capacity (TAC) level in NHDF cells significantly depended on the type of graphene oxide applied in the experiment and was significantly reduced after GO and GOS exposure in comparison to control cells (Table 1, Table S1, and Figure 1c), where it was the highest. For this oxidative stress marker, the time had no impact on its level in any of the experimental groups: TAC levels were the same in GO- and GOS-exposed NHDF cells one hour after the exposure.

Malondialdehyde (MDA) concentration depended on the time of exposure, on the graphene oxide type, and on the interaction between these two factors. After the first hour of the experiment, MDA concentrations were significantly higher in GO-exposed than in GOS-exposed and control NHDF cells. After 24 and 48 h, MDA concentrations were significantly higher in GOS-exposed NHDF cells in comparison to control and GO groups’ cells (Table 1, Table S1, and Figure 1d).

#### 3.1.2. A549 Cell Line

The levels of oxidative stress markers measured in the A549 (adenocarcinomic human alveolar basal epithelial) cells were similar to those detected in NHDF cells (Table 1 and Table S1).

**Figure 2:**
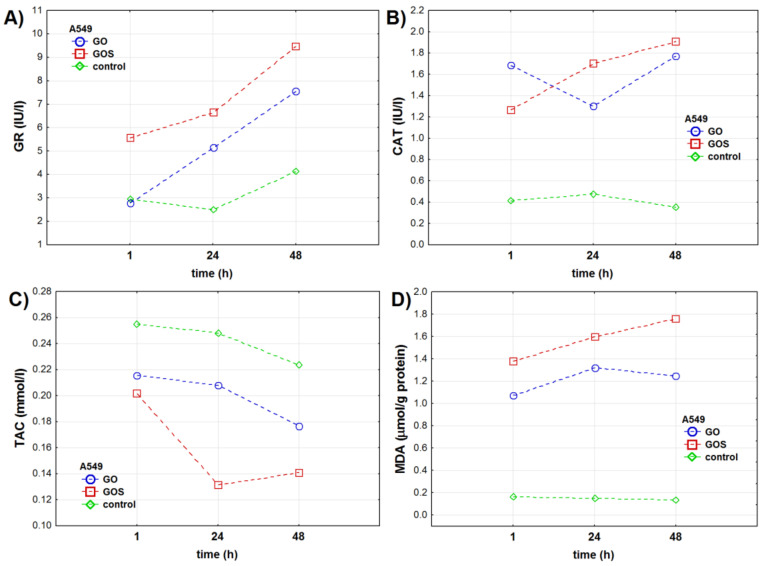
Oxidative stress markers in the in the A549 (adenocarcinomic human alveolar basal epithelial cells) cell line measured 1, 24, and 48 h after exposure to ultrapure (GO) or manganese ions-contaminated graphene oxide (GOS): (**A**) glutathione reductase (GR) activity (IU/L), (**B**) catalase (CAT) activity (IU/L), (**C**) total antioxidant capacity (TAC) (mmol/L), (**D**) malondialdehyde (MDA) concentration (μmol/g protein). The markers present mean value of the analyzed parameter.

GR activity measured in A549 cells significantly depended on the time of exposure and combination of graphene type used and time of the exposure. Nevertheless, the results of ANOVA analysis presented in the Table 1 show that the type of graphene oxide itself did not influence GR activity. GO and GOS exposure increased GR activity in A549 cells, when compared to the control cells. After 48 h, GR activity was significantly higher in both experimental groups (Figure 2a). GO and GOS exposure increased GR activity after 24 and 48 h when compared to GR activity in control cells in respective time points. Moreover, 24 and 48 h after GO exposure, GR levels were 2 times and 3 times higher when compared to GR activity after 1 and 24 h.

CAT activity in A549 cells depended on the time of exposure, type of graphene oxide itself, and a combination of both factors (Table 1 and Table S1). CAT activity significantly increased in GO and GOS exposed cells when compared to control A549 cells (Figure 2b). Moreover, CAT activity was significantly different in GO- and GOS-exposed A549 cells at all time points of the experiment. One hour after exposure, CAT activity was significantly higher in GO-exposed cells when compared to control and GOS-exposed A549 cells. After 24 and 48 h, CAT activity increased in GOS-exposed cells.

TAC levels in A549 cells depended on the type of graphene oxide, time of exposure, and combination of these two parameters (Table 1, Table S1, and Figure 2c). TAC level was significantly lower at all studied times in GO- and GOS-exposed cells when compared to control A549 cells. TAC levels in GO- and GOS-exposed cells were similar only 1 h after the exposure.

Lipid peroxidation measured with MDA significantly depended on the time of exposure (differently for each type of graphene oxide) but not on the graphene oxide itself (Table 1 and Table S1). In the A549 cells, MDA concentration was up to 8–12 times higher in GO- and GOS-exposed cells when compared to control A549 cells at all experimental time points (Figure 2d).

### 3.2. Pro-Inflammatory Cytokines in Cells Exposed to Graphene Oxide Manganese Ion Contaminated (GO + Mn^2+^, GOS) and Graphene Oxide Ultrapure (GO) for 1, 24, and 48 h

#### 3.2.1. NHDF Cell Line

Cytokines levels were related to the exposure time, the graphene oxide type, and the combination of these parameters (Table 2).

**Figure 3:**
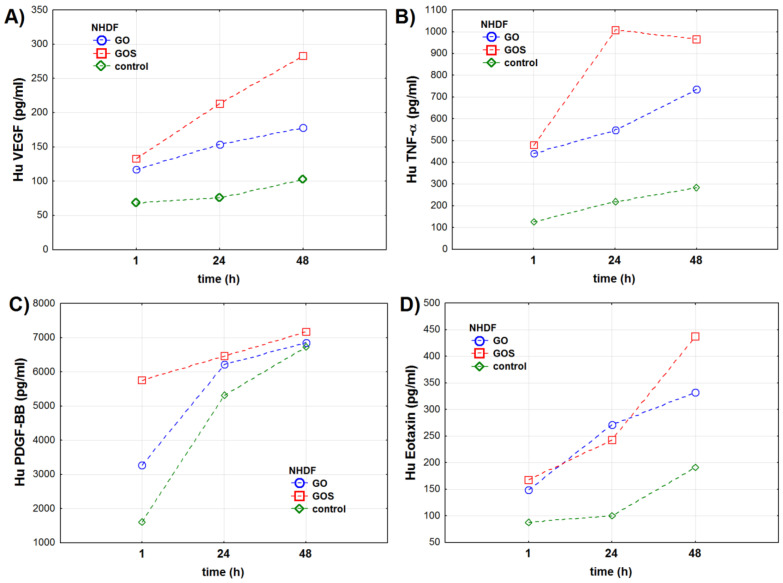
Cytokines’ concentrations (pg/mL) in the NHDF (normal human dermal fibroblasts) cell line measured 1, 24, and 48 h after exposure to ultrapure (GO) or manganese ions-contaminated graphene oxide (GOS): (**A**) vascular endothelial growing factor (VEGF), (**B**) tumor necrosis factor-alpha (TNF-α), (**C**) platelet-derived growth factor-BB (PDGF-BB), (**D**) eotaxin. The markers present mean value of the analyzed parameter.

The results show that VEGF (vascular endothelial growing factor) level in the NHDF cells was related to the type of graphene oxide, the exposure time, and the combination of these two factors (Table 2). VEGF levels significantly increased after GO and GOS exposure when compared to control cells. Moreover, VEGF levels increased in time in GO- and GOS-exposed cells and in control cells (Table 2, Table S2, and Figure 3a). The analysis showed that GOS exposure had the strongest impact on VEGF levels in time when compared to control and GO-exposed cells.

TNF-α (tumor necrosis factor-alpha) levels also depended on the exposure time, type of graphene, and combination of these two factors. TNF-α levels in control and GO-exposed cells were significantly higher after 1, 24, and 48 h (Table 2 and Table S2). For GOS-exposed cells, the highest level of TNF-α was observed after 24 h when compared to its levels after 1 and 48 h exposure. TNF-α levels were the highest in GO- and GOS-exposed cells after 24 and 48 h (Figure 3b).

Similar to TNF-α, PDGF-BB (platelet-derived growth factor-BB) levels were related to the exposure time, type of graphene used, and combination of those factors (Table 2). Again, the highest level of PDGF-BB was recorded for GOS-exposed cells. The lowest levels of PDGF-BB were observed in control NHDF cells 1 h into the experiment. The levels of PDGF-BB significantly increased in the 24th and 48th hours of the experiment, especially in control and GO-exposed cells (Table 2 and Figure 3c).

Eotaxin levels were related to the exposure time, type of graphene oxide, and combination of these two factors (Table S2). Eotaxin levels were the same after 1 h and after 24 h of the experiment in control NHDF cells (Table S2). Moreover, eotaxin levels of GO- and GOS-exposed cells in the 1st and 24th hours of the experiment were also the same (Table S2 and Figure 3d). We noticed significant differences in eotaxin levels for GO- and GOS-exposed cells in the 48th hour of the experiment.

#### 3.2.2. A549 Cell Line

In the A549 cells, the levels of all analyzed cytokines were related to the exposure time, the type of graphene oxide used, and the combination of these parameters (Table 2).

**Figure 4:**
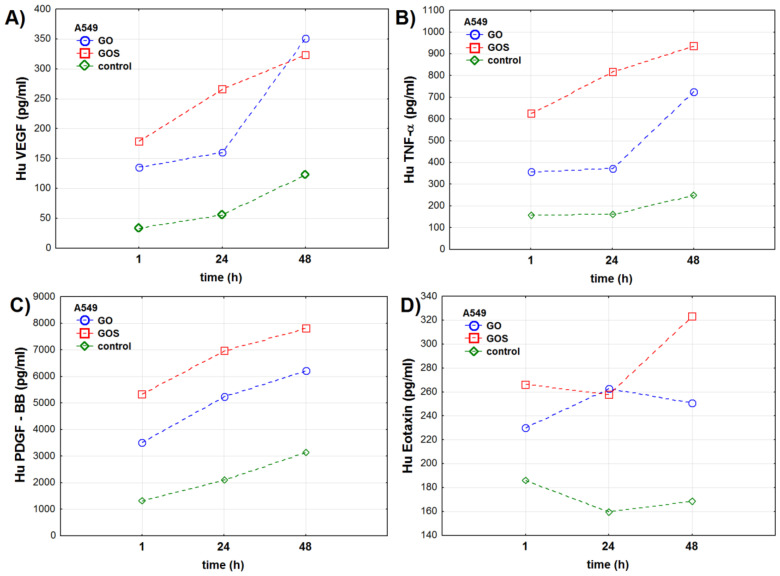
Cytokines’ concentration (pg/mL) in the A549 (adenocarcinomic human alveolar basal epithelial cells) cell line measured 1, 24, and 48 h after exposure to ultrapure (GO) or manganese ions-contaminated graphene oxide (GOS): (**A**) vascular endothelial growing factor (VEGF), (**B**) tumor necrosis factor-alpha (TNF-α), (**C**) platelet-derived growth factor-BB (PDGF-BB), (**D**) eotaxin. The markers present mean value of the analyzed parameter.

VEGF levels significantly increased over the time of the experiment, reaching the highest values after 48 h in all groups of A549 cells. The level of this cytokine was significantly lower in the control cells when compared to GO- and GOS-exposed cells. GOS exposure strongly stimulated secretion of VEGF and its level was the highest when compared to GO-exposed and control A549 cells (Table 2, Table S2, and Figure 4a).

TNF-α levels were the highest in the GOS-exposed cells at every time point of the experiment. The lowest levels of TNF-α were recorded for control cells during the whole experiment. The TNF-α levels after the 1st and the 24th h of the experiment were the same for control and GO-exposed A549 cells (Table 2, Table S2, and Figure 4b).

PDGF-BB levels increased during the time of experiment and the time profiles of PDGF-BB were similar in all tested cells: The lowest level of this cytokine was observed in the 1st hour of the experiment and then it gradually increased up to the 48th hour of the experiment (Table 2, Table S2, and Figure 4c). The lowest levels of this cytokine were recorded for the control cells, whereas the highest were for GOS-exposed A549 cells.

Eotaxin level was the lowest in the control A549 cells (Table 2). Exposure to GO and GOS significantly stimulated the increase in this cytokine level when compared to control cells at all time points of the experiment (Table 2 and Table S2). The levels of eotaxin in GO- and GOS-exposed cells were different after the 1st and 48th hours of the experiment (Table S2 and Figure 4d).

## 4. Discussion

This work investigated the early answer of the normal human dermal fibroblasts (NHDF) and adenocarcinomic human alveolar basal epithelial cells (A549) to the presence of ultrapure (GO) and Mn^2+^-contaminated graphene oxide (GOS). We compared oxidative stress markers and cytokines’ levels in the NHDF and A459 cells at the selected time points: 1, 24, and 48 h after exposure to two types of graphene oxide.

The analyses of oxidative stress markers and cytokines in the NHDF cell line showed that: (1) Three out of four oxidative stress markers (glutathione reductase (GR) and catalase (CAT) activity and malondialdehyde (MDA) concentration) depended on the exposure time differently for each graphene oxide type; (2) total antioxidant capacity (TAC) depended only on the type of graphene oxide used in the experiment; (3) there were no significant differences between GO and GOS short-time effects on TAC levels; (4) GO and GOS exposure significantly enhanced lipid peroxidation, measured with MDA, that increased in time; (5) the levels of selected cytokines (vascular endothelial growing factor (VEGF), tumor necrosis factor-alpha (TNF-α), platelet-derived growth factor-BB (PDGF-BB), and eotaxin) depended on the time of exposure, type of graphene oxide, and interaction between those two factors; and (6) both types of graphene oxide significantly induced oxidative stress in comparison to the control cells. Nevertheless, the biological answer assessed with selected oxidative stress markers and cytokines was stronger in GOS-exposed NHDF cells.

The analyses of oxidative stress markers and cytokines in the A549 cell line showed that: (1) The antioxidative response of A549 cells, measured with GR, TAC, CAT, and MDA, was related to the exposure time and graphene oxide type. However, TAC level and CAT activity depended on the graphene oxide type, the exposure time, and the combination between these two factors. (2) GR activity and MDA concentration depended on the exposure time and combination between the exposure time and graphene oxide type, but not on the type of graphene oxide itself. (3) GR activity in GO/GOS-exposed cells increased over the time of the experiment. (4) Cytokines’ levels increased after exposure to GO or GOS.

We made three joint observations for NHDF and A549 cell lines: (1) Exposure time and interaction between exposure time and graphene oxide type induced stronger antioxidative answer than graphene oxide type itself; (2) Mn^2+^-contaminated graphene oxide induced higher levels of oxidative stress than ultrapure graphene oxide when compared to control cells and the overall reaction to oxidative stress, measured with CAT activity, TAC, and MDA concentration, was similar in both cell lines; and (3) the increase in cytokines’ levels was comparable in both cell lines.

The only notable difference between the tested cell lines in their response to graphene oxide exposure was found for GR activity. It showed a different pattern in each cell line: It decreased in time (1, 24, and 48 h) in NHDF, but increased in A549 cell line.

### 4.1. Oxidative Stress Markers’ Response to Graphene Oxide Exposure

The response of selected oxidative stress markers to graphene oxide exposure depended on exposure time and GO type in both cell lines. The degree and the trend of the changes were different for the A549 and NHDF cells. A549 cell line showed, in contrast to the NHDF cell line, higher GR activity when exposed to GO and GOS. Additionally, it was directly proportional to the exposure time. The activity of GR in GOS-exposed NHDF cells decreased in time, with the lowest activity measured after 48 h of exposure. GR catalyzes and converts oxidized glutathione (GSSG) to reduced glutathione (GSH) in an NADPH-dependent manner, managing the glutathione pool that is necessary to maintain functional proteins under normal and stress conditions [29]. The decreasing-in-time activity of the glutathione-regenerating enzyme (here GR) after GOS exposure may suggest the reduced pool of GSH in the NHDF cells (but not in A549 cancer cells). However, this should be supported by additional analysis, which was not included in the study design. We concluded that, in terms of GR activity, the time of short-term exposure, the interaction between time and type of graphene oxide used, and the type of cells selected for the experiment is more important than the type of graphene oxide used itself. Chang et al. [5] reported that GO exposure promotes the production of high levels of reactive oxygen species (ROS). Their study showed that the levels of ROS depended on the exposure time and graphene oxide concentration. This indirectly confirms our findings because GR expression and activity depend on ROS levels in a cell: the higher ROS concentration in the cell, the higher GR expression and activity that enable it to neutralize the negative effects of ROS [5].

Graphene-family nanomaterials change the structural stability of the plasmalemma and cause physical injuries to the cell membranes. In consequence, damaged cell membranes promote damage in cell structures and enhance oxidative stress [30]. We found that GO and GOS exposure induced higher CAT activity both in NHDF and A549 cells when compared to control cells. In the GOS-exposed A459 cells, CAT activity increased in time, while in the GOS-exposed NHDF cells, it also increased but remained at a comparable level after 24 and 48 h of the experiment. We observed no differences in CAT activity between GO- and GOS-exposed NHDF cells after 24 h of the experiment. CAT is one of the major enzymes of the first-line defence system against oxidative toxicity [31]. In our study, CAT activity significantly increased after 1, 24, and 48 h of GO and GOS exposure in NHDF and A549 cells when compared to the respective controls. GO and GOS exposure stimulated CAT activity, suggesting that CAT efficiently removed H_2_O_2_. Wei and Ge [32] reported that CAT activity and conformation are related to GO concentration. They found a dramatic decrease in CAT activity isolated from bovine liver 1 h after exposure to low doses of GO. GO contains many reactive oxygen functional groups that may directly interfere with the oxyferryl electron transfer in catalase, which subsequently leads to decreased activity [33,34]. This mechanism, however, cannot be considered as an explanation for our results. Firstly, in our experiment, the elements that were directly exposed to GO/GOS were the cell membranes and other cell structures, not the enzymes themselves. Secondly, we suggest that exposure to graphene oxide induces OS, which is indirectly confirmed by increased CAT activity and TAC level.

Total antioxidant capacity is one of the most complex oxidative stress markers. It represents the sum of all antioxidants present in the examined cell or tissue [35]. Our research showed that exposure to GO and GOS decreased TAC levels both in NHDF and A549 cells. TAC level in normal and cancer cells was significantly decreased already in the first hour of the experiment in comparison to the control cells, but this change was higher in the NHDF cells when compared to A549 cells. In the NHDF cells, TAC level was only susceptible to the graphene type. The GO type was the main factor affecting TAC, which helps to evaluate the overall oxidative stress status in normal cells. The impact of GO and GOS on NHDF and A549 TAC levels was similar after the first hour and then, after 24 and 48 h, the cells reacted differently. In cancer A549 cells, TAC levels after the first hour of exposure were the same in GO- and GOS-exposed cells. It means that both types of graphene had a similar negative effect on the tested cell lines, but it changed after 24 and 48 h. TAC levels in A549 cells depended on the exposure time, graphene type, and their interaction. The changes were more dynamic in the GOS-exposed cells where the suppressing impact was observed after 24 h. This agrees with literature data that show that graphene oxide toxicity in A549 cells depends on the exposure time [5]. Both A549 and NHDF cells were directly exposed to graphene oxide derivatives. Thus, a decrease in TAC level somehow was expected. But we found that the cells’ answer depended on their nature. We suggest that A549 cells are more resistant since the changes in TAC level in the GO-exposed cells showed only after 48 h. This may be connected with the altered biology of cancer cells and their higher adjustability to environmental factors [36,37].

Our results showed higher MDA concentration after GO and GOS exposure when compared to control cells. Moreover, we found that GOS exposure induced the highest levels of MDA in both tested cell lines. A similar influence of graphene oxide on MDA levels was observed by Wang et al. [8] in human multiple myeloma RPMI 8226 cells. The authors found that graphene oxide exposure significantly increased intracellular MDA production [8]. Those results, others’, and ours indicate that all types of cells exposed to the graphene oxide, despite their character, experience oxidative stress and subsequently enhanced lipid peroxidation. Enhanced MDA production seems to be the primary cytotoxic mechanism that can be observed after GO exposure. We suggest that the clinical significance of our results should be investigated.

Based on the results for NHDF and A549 cell lines exposed to different types of graphene oxide, we concluded that GOS has a significantly worse effect on the oxidative stress markers’ levels. We noticed that, in general, the graphene oxide toxicity depended on the cell culture exposure time, which was confirmed by literature data [5,38,39], and the type of graphene oxide—ultrapure or Mn^2+^-contaminated—as manganese contamination might be a reason for the increased negative influence of GOS. Manganese is an essential mineral that is found at low levels in virtually all diets. Regardless of intake, animals generally maintain stable tissue Mn^2+^ levels as a result of homeostatic mechanisms [40]. Manganese ion impurities in nanoparticles, at the concentration applied in this study, should not have any significant influence on the physiological process. Nevertheless, in vitro conditions showed that the cytotoxicity of graphene-derived materials remains controversial and is dependent on a series of physical-chemical parameters [41].

### 4.2. Cytokines’ Response to Graphene Oxide Exposure

Vascular endothelial growing factor (VEGF) is a protein produced by many types of cells, e.g., cancer cells, macrophages, platelets, and keratinocytes. It is involved mainly in angiogenesis and apoptosis prevention [42]. Our research showed that both cell lines produced significantly higher VEGF levels than respective control cells after exposure to GO or GOS. Morevoer, we found that GOS exposure promoted the highest VEGF production in the cells. Other studies mention that higher cell VEGF concentrations may occur in insufficient oxygen supply conditions [43], in vitro cultures, or insufficient serum level [44]. Hu et al. demonstrated that graphene was able to bind to serum in the culture fluid and, therefore, was responsible for the nutrients’ deficiency in the studied cells [38]. Other studies proved that graphene coats the cells and causes their hypoxia [45]. This last mechanism can explain why we observed the increased VEGF levels in GO- and GOS-exposed cells.

A high concentration of tumor necrosis factor-alpha (TNF-α) promotes necrotic changes in the cell and its death by increasing free radicals’ levels in the cell [46]. Our study showed that the TNF-α levels were significantly higher in GO- and GOS-exposed cells than in the control cells of both tested lines. Again, GOS exposure was connected with the highest concentrations of this cytokine. Ou et al. mention that various forms of graphene, including graphene oxide, may be responsible for increased TNF-α levels resulting from cell damage or anti-inflammatory response induction [45].

The platelet-derived growth factor-BB regulates cell growth and division. In the case of platelets, PDGF-BB is one of the key substances that take part in natural wound regeneration [47]. Our study showed that the PDGF-BB level was about two times higher in the NHDF cells exposed to GO and GOS than in the control cells after the first hour of exposure. At the 24-h and 48-h time points, the PDGF-BB level in control cells was similar to its levels in GO- and GOS-exposed cells, but GOS-exposed cells still produced higher levels of this cytokine. In A549 cells, the initial situation was similar. The control cells produced lower levels of PDGF-BB than GO- and GOS-exposed cells. However, the difference between the control cells and those exposed to graphene oxide was substantial at the 24-h and 48-h time points. Moreover, in this case, exposure to GOS was related to the highest levels of PDGF-BB. High concentrations of this cytokine in the exposed cells may result from mechanical damage to the cell caused by graphene oxide because, as Oefner et al. reported, this protein plays a key role in wound regeneration [47]. However, the upregulation in the PDGF-BB expression has been linked to neoplastic transformation and cancer metastasis [48]. Additionally, changes and dysregulation of PDGF levels, often accompanied by changes in VEGF levels, as we reported for our experiment, are associated with various malignancies [49]. Chronic dysregulation of these two cytokines may lead to cancer development, which may partly explain its higher concentrations in A549 cells in comparison with NHDF cells.

Eotaxin is produced mainly in pulmonary respiratory epithelial cells during inflammation or allergic reactions [46]. Our study showed that in both tested cell lines eotaxin levels were 1–1.5 times higher in cells exposed to GO and GOS than in the control cells. The highest concentrations of eotaxin were observed for the A549 cells, both in the graphene oxide-exposed and control cells. That may have resulted from the nature of the A549 cell line. A549 is a tumor lung epithelial line and, as already mentioned, eotaxin is mainly produced by lung epithelial cells [50]. Our study also showed that exotoxin levels increased over time in NHDF cells. That might be related to the physicochemical properties of the tested materials and the biology of selected cell lines. This finding coincides with the results of Roberts et al., whose study showed that eotaxin expression increased during the first day of exposure for all tested graphite nanoplates (derivative of the graphene nanoparticles’ family) [51].

In both analysed cells, NHDF and A549, the levels of cytokines were related to the exposure time, graphene oxide type, and interaction between those factors. Analysing the general profiles of tested cytokines, we noted higher levels of VEGF and eotaxin in the A549 cells and higher TNF-α levels in the NHDF cells. As for PDGF-BB, we noted similar levels of this cytokine for both tested cell lines. Nevertheless, taking into consideration the profiles of cytokines, both types of cells showed similar susceptibility to graphene oxide, both ultrapure and manganese ions-contaminated, after short-time exposure. However, manganese-contaminated graphene oxide was related to the highest concentrations of cytokines. We hypothesized that GOS has more negative effects on tested cells due to its contamination. At the cellular level, manganese ions can cause mitochondrial dysfunction and, thus, the oxidative stress, can change cellular proteins’ conformation, can disturb apoptosis, and can interact with other metals [52].

## 5. Conclusions

In NHDF (normal human dermal fibroblasts) and A549 (adenocarcinomic human alveolar basal epithelial cells) cell cultures, the answer to ultrapure (GO) and Mn^2+^-contaminated (GOS) graphene oxide exposure measured with oxidative stress markers was more related to the exposure time than to the type of graphene oxide. Independently, in the same types of cells, the cytokines’ levels were related to the exposure time as well as to the GO type. Short-time exposure to Mn^2+^-contaminated GO induced the stronger response of oxidative stress markers but this increase did not collapse the antioxidative systems of analyzed cells. Increased levels of inflammatory cytokines after GO- and Mn^2+^-contaminated GO exposure were similar both in NHDF and cancer A549 cells, which shows that cytotoxicity of the studied graphene-derived materials triggers comparable physiological answers in in vitro conditions.

## Figures and Tables

**Table 1:** Oxidative stress markers’ levels in NHDF (normal human dermal fibroblasts) and A549 (adenocarcinomic human alveolar basal epithelial cells) cell line measured 1, 24, and 48 h after exposure to ultrapure (GO) or manganese ions-contaminated graphene oxide (GOS). Categorical factors: time (time passed from exposition to GO/GOS to samples’ collection), factor (GO or GOS vs. control group); int. (interaction between time and type of factor (GO vs. GOS)). The results are presented as mean ± standard deviation. Statistical significance was set at a *p* < 0.05.

Oxidative Stress Marker	Group	NHDF Cell Line						A549 Cell Line					
		1 h	24 h	48 h	p_time_	p_factor_	p_int._	1 h	24 h	48 h	p_time_	p_factor_	p_int._
GR (IU/l)	control	0.96 ± 0.04	0.74 ± 0.01	1.53 ± 0.01	<0.001	<0.001	<0.001	2.95 ± 0.02	2.49 ± 0.01	4.12 ± 0.15	<0.001	–	<0.001
	GO	1.27 ± 0.02	1.67 ± 0.01	1.87 ± 0.01				2.78 ± 0.01	5.14 ± 0.05	7.54 ± 0.03			
	GOS	1.62 ± 0.01	1.18 ± 0.04	0.57 ± 0.02				5.56 ± 0.04	6.64 ± 0.02	9.47 ± 0.02			
CAT (IU/l)	control	0.32 ± 0.02	0.19 ± 0.02	0.17 ± 0.03	<0.001	–	<0.001	0.41 ± 0.11	0.48 ± 0.04	0.35 ± 0.03	<0.001	<0.001	<0.001
	GO	1.13 ± 0.02	1.28 ± 0.02	1.13 ± 0.02				1.68 ± 0.01	1.30 ± 0.03	1.77 ± 0.02			
	GOS	1.17 ± 0.02	1.31 ± 0.03	1.31 ± 0.04				1.27 ± 0.02	1.70 ± 0.05	1.91 ± 0.05			
TAC (mmol/l)	control	0.24 ± 0.01	0.25 ± 0.03	0.25 ± 0.05	0.192	<0.001	0.072	0.25 ± 0.02	0.25 ± 0.02	0.22 ± 0.01	<0.001	< 0.001	<0.001
	GO	0.14 ± 0.02	0.16 ± 0.01	0.14 ± 0.02				0.22 ± 0.03	0.21 ± 0.01	0.18 ± 0.01			
	GOS	0.14 ± 0.01	0.13 ± 0.01	0.15 ± 0.03				0.20 ± 0.02	0.13 ± 0.01	0.14 ± 0.01			
MDA (μmol/g protein)	control	0.30 ± 0.08	0.16 ± 0.02	0.19 ± 0.02	<0.001	<0.001	<0.001	0.16 ± 0.02	0.15 ± 0.02	0.14 ± 0.02	<0.001	–	<0.001
	GO	1.60 ± 0.06	1.92 ± 0.02	1.63 ± 0.08				1.07 ± 0.03	1.32 ± 0.05	1.24 ± 0.08			
	GOS	1.40 ± 0.01	2.17 ± 0.02	2.39 ± 0.05				1.38 ± 0.04	1.60 ± 0.02	1.76 ± 0.08			

Abbreviations: A549, adenocarcinomic human alveolar basal epithelial cells cell line; CAT, catalase activity; GR, glutathione reductase activity; GO, ultrapure graphene oxide; GOS, graphene oxide contaminated with manganese ions; MDA, malondialdehyde concentration; NHDF, normal human dermal fibroblasts cell line; TAC, total antioxidant capacity.

**Table 2:** Cytokines levels in NHDF (normal human dermal fibroblasts) and in A549 (adenocarcinomic human alveolar basal epithelial cells) cell line measured 1, 24, and 48 h after exposure to ultrapure (GO) or manganese ions-contaminated graphene oxide (GOS). Categorical factors: time (time passed from exposition to GO/GOS to samples collection), factor (GO or GOS vs. control group), int. (interaction between time and type of factor (GO vs. GOS)). The results are presented as mean ± standard deviation. Statistical significance was set at a *p* < 0.05.

Cytokines Concentration [pg/mL]	Group	NHDF Cell Line						A549 Cell Line					
		1 h	24 h	48 h	p_time_	p_factor_	p_int._	1 h	24 h	48 h	p_time_	p_factor_	p_int._
VEGF	Control	68.0 ± 0.9	75.8 ± 0.7	102.1 ± 1.3	<0.001	<0.001	<0.001	32.8 ± 0.6	55.3 ± 1.0	123.4 ± 1.3	<0.001	<0.001	<0.001
	GO	117.2 ± 0.6	153.7 ± 1.0	178.3 ± 2.0				135.9 ± 1.1	160.2 ± 0.5	351.0 ± 0.9			
	GOS	132.9 ± 1.8	213.7 ± 0.8	283.3 ± 1.4				178.8 ± 0.8	265.8 ± 0.9	323.9 ± 0.7			
TNF-α	Control	125.5 ± 16.1	218.8 ± 26.1	283.5 ± 18.5	<0.001	<0.001	<0.001	158.3 ± 11.8	161.3 ± 9.9	248.7 ± 29.8	<0.001	<0.001	<0.001
	GO	441.3 ± 37.0	550.2 ± 45.7	736.3 ± 28.7				357.5 ± 22.2	370.2 ± 38.2	726.3 ± 15.5			
	GOS	477.7 ± 47.7	1008.5 ± 21.1	966.8 ± 55.8				625.5 ± 11.6	817.0 ± 35.5	934.5 ± 20.9			
PDGF-BB	Control	1606.7 ± 51.9	5306.3 ± 67.7	6725.0 ± 152.5	<0.001	<0.001	<0.001	1304.8 ± 7.7	2117.8 ± 12.0	3134.8 ± 25.4	<0.001	<0.001	<0.001
	GO	3267.8 ± 84.1	6212.5 ± 101.6	6864.3 ± 43.6				3512.0 ± 9.2	5248.8 ± 19.8	6206.3 ± 142.3			
	GOS	5746.0 ± 75.1	6462.7 ± 124.7	7171.8 ± 96.9				5334.8 ± 103.1	6954.8 ± 93.8	7807.7 ± 74.3			
Eotaxin	Control	88.0 ± 10.1	99.7 ± 16.2	191.0 ± 22.0	<0.001	<0.001	<0.001	186.2 ± 10.8	159.8 ± 12.9	169.3 ± 19.4	<0.01	<0.001	<0.001
	GO	149.0 ± 41.2	271.3 ± 34.6	331.7 ± 36.0				229.7 ± 21.1	262.8 ± 22.8	251.2 ± 6.2			
	GOS	167.7 ± 18.0	242.3 ± 34.9	437.0 ± 39.2				266.3 ± 25.6	257.8 ± 28.2	323.0 ± 3.2			

Abbreviations: A549, adenocarcinomic human alveolar basal epithelial cells; GO, ultrapure graphene oxide; GOS, graphene oxide contaminated with manganese ions; NHDF, normal human dermal fibroblasts cell line; PDGF-BB, platelet-derived growth factor-BB; TNF-α, tumor necrosis factor-alpha; VEGF, vascular endothelial growing factor.

## Data Availability

The data are available after contact with the corresponding author.

## Supplementary Materials

The following are available online at https://www.mdpi.com/article/10.3390/antiox10050765/s1. Table S1. Multiple comparisons in contrast analysis for oxidative stress markers measured in NHDF (normal human dermal fibroblasts) and A549 (adenocarcinomic human alveolar basal epithelial cells) cell line 1, 24, and 48 h after exposure to ultrapure (GO) or manganese ions-contaminated graphene oxide (GOS). Comparison between study groups for NHDF cell line: control, GO0-, and GOS-exposed. Statistical significance was set at a *p* < 0.05. Table S2. Multiple comparisons in contrast analysis for cytokines’ levels measured in NHDF (normal human dermal fibroblasts) and in A549 (adenocarcinomic human alveolar basal epithelial cells) cell line 1, 24, and 48 h after exposure to ultrapure (GO) or manganese ions-contaminated graphene oxide (GOS). Comparison between study groups for NHDF cell line: control, GO-, and GOS-exposed. Statistical significance was set at a *p* < 0.05.
