# Supplementary material for: Graphene Oxide Normal (GO + Mn2+) and Ultrapure: Short-Term Impact on Selected Antioxidant Stress Markers and Cytokines in NHDF and A549 Cell Lines
Source: Antioxidants (Basel). 2021 May 11;10(5):765. doi: 10.3390/antiox10050765 (PMC8151183; doi:10.3390/antiox10050765)
Supplement: Supplementary file 1 [file antioxidants-10-00765-s001.zip › antioxidants-1139927-supplementary.pdf]

Table S1. Multiple comparisons in contrast analysis for oxidative stress markers measured in NHDF (normal human dermal fibroblasts) and A549 (adenocarcinomic human alveolar basal epithelial cells) cell line 1, 24 and 48 h after exposure to ultrapure (GO) or manganese ions contaminated graphene oxide (GOS). Comparison between study groups for NHDF cell line: control, GO and GOS exposed. Statistical significance was set at a $p < 0.05$.

Oxidative Stress Marker	Comparison	NHDF Cell Line			A549 Cell Line		
		1 h	24 h	48 h	1 h	24 h	48 h
GR (IU/l)	$p_{control\ vs.\ GO}$	< 0.001	< 0.001	< 0.001	< 0.001	< 0.001	< 0.001
	$p_{control\ vs.\ GOS}$	< 0.001	< 0.001	< 0.001	< 0.001	< 0.001	< 0.001
	$p_{GOS\ vs.\ GO}$	< 0.001	< 0.001	< 0.001	< 0.001	< 0.001	< 0.001
CAT (IU/l)	$p_{control\ vs.\ GO}$	< 0.001	< 0.001	< 0.001	< 0.001	< 0.001	< 0.001
	$p_{control\ vs.\ GOS}$	< 0.001	< 0.001	< 0.001	< 0.001	< 0.001	< 0.001
	$p_{GOS\ vs.\ GO}$	< 0.001	0.099	< 0.001	< 0.001	< 0.001	< 0.001
TAC (mmol/l)	$p_{control\ vs.\ GO}$	< 0.001	< 0.001	< 0.001	< 0.001	< 0.001	< 0.001
	$p_{control\ vs.\ GOS}$	< 0.001	< 0.001	< 0.001	< 0.001	< 0.001	< 0.001
	$p_{GOS\ vs.\ GO}$	0.704	< 0.001	< 0.001	0.100	< 0.001	< 0.001
MDA (μmol/g protein)	$p_{control\ vs.\ GO}$	< 0.001	< 0.001	< 0.001	< 0.001	< 0.001	< 0.001
	$p_{control\ vs.\ GOS}$	< 0.001	< 0.001	< 0.001	< 0.001	< 0.001	< 0.001
	$p_{GOS\ vs.\ GO}$	< 0.001	< 0.001	< 0.001	< 0.001	< 0.001	< 0.001

Abbreviations: A549 – adenocarcinomic human alveolar basal epithelial cells cell line, CAT – catalase activity, GR – glutathione reductase activity, GO – ultrapure graphene oxide, GOS – graphene oxide contaminated with manganese ions, MDA – malondialdehyde concentration, NHDF – normal human dermal fibroblasts cell line, TAC – total antioxidant capacity.

Table S2. Multiple comparisons in contrast analysis for cytokines levels measured in NHDF (normal human dermal fibroblasts) and in A549 (adenocarcinomic human alveolar basal epithelial cells) cell line 1, 24 and 48 h after exposure to ultrapure (GO) or manganese ions contaminated graphene oxide (GOS). Comparison between study groups for NHDF cell line: control, GO and GOS exposed. Statistical significance was set at a $p < 0.05$.

Cytokine Concentration [pg/mL]	Comparison	NHDF Cell Line			A549 Cell Line		
		1 h	24 h	48 h	1 h	24 h	48 h
VEGF	$p_{control\ vs.\ GO}$	< 0.001	< 0.001	< 0.001	< 0.001	< 0.001	< 0.001
	$p_{control\ vs.\ GOS}$	< 0.001	< 0.001	< 0.001	< 0.001	< 0.001	< 0.001
	$p_{GOS\ vs.\ GO}$	< 0.001	< 0.001	< 0.001	< 0.001	< 0.001	< 0.001
TNF-α	$p_{control\ vs.\ GO}$	< 0.001	< 0.001	< 0.001	< 0.001	< 0.001	< 0.001
	$p_{control\ vs.\ GOS}$	< 0.001	< 0.001	< 0.001	< 0.001	< 0.001	< 0.001
	$p_{GOS\ vs.\ GO}$	0.101	< 0.001	< 0.001	< 0.001	< 0.001	< 0.001
PDGF-BB	$p_{control\ vs.\ GO}$	< 0.001	< 0.001	< 0.05	< 0.001	< 0.001	< 0.001
	$p_{control\ vs.\ GOS}$	< 0.001	< 0.001	< 0.001	< 0.001	< 0.001	< 0.001
	$p_{GOS\ vs.\ GO}$	0.704	< 0.001	< 0.001	0.100	< 0.001	< 0.001
Eotaxin	$p_{control\ vs.\ GO}$	< 0.01	< 0.001	< 0.001	< 0.01	< 0.001	< 0.001
	$p_{control\ vs.\ GOS}$	< 0.001	< 0.001	< 0.001	< 0.001	< 0.001	< 0.001
	$p_{GOS\ vs.\ GO}$	0.243	0.113	< 0.001	< 0.01	0.702	< 0.001

Abbreviations: A549 – adenocarcinomic human alveolar basal epithelial cells, GO – ultrapure graphene oxide, GOS – graphene oxide contaminated with manganese ions, NHDF – normal human dermal fibroblasts cell line, PDGF-BB – platelet-derived growth factor-BB, TNF-α – tumor necrosis factor-alpha, VEGF – vascular endothelial growing factor.
